# Chromatin Remodelers in the 3D Nuclear Compartment

**DOI:** 10.3389/fgene.2020.600615

**Published:** 2020-11-03

**Authors:** Mauro Magaña-Acosta, Viviana Valadez-Graham

**Affiliations:** Departamento de Genética del Desarrollo y Fisiología Molecular, Instituto de Biotecnología, Universidad Nacional Autónoma de México, Cuernavaca, Mexico

**Keywords:** chromatin remodeling, 3D organization, chromatin structure, architectural proteins, ATP-dependent remodeling complexes

## Abstract

Chromatin remodeling complexes (CRCs) use ATP hydrolysis to maintain correct expression profiles, chromatin stability, and inherited epigenetic states. More than 20 CRCs have been described to date, which encompass four large families defined by their ATPase subunits. These complexes and their subunits are conserved from yeast to humans through evolution. Their activities depend on their catalytic subunits which through ATP hydrolysis provide the energy necessary to fulfill cellular functions such as gene transcription, DNA repair, and transposon silencing. These activities take place at the first levels of chromatin compaction, and CRCs have been recognized as essential elements of chromatin dynamics. Recent studies have demonstrated an important role for these complexes in the maintenance of higher order chromatin structure. In this review, we present an overview of the organization of the genome within the cell nucleus, the different levels of chromatin compaction, and importance of the architectural proteins, and discuss the role of CRCs and how their functions contribute to the dynamics of the 3D genome organization.

## Chromatin Structure and Organization

Eukaryotic DNA is compartmentalized into hierarchically organized levels within the nuclear space. To achieve this, the genetic material interacts with diverse proteins in a non-random 3D array that helps to form a complex called chromatin. This DNA–protein complex functions to maintain the architecture of the genome, stabilize it, and regulate the accessibility of the transcriptional machinery to certain regions, while maintaining other regions silenced (van Bortle and Corces, 2012; Fraser et al., 2015; Bonev and Cavalli, 2016; Rowley and Corces, 2018; Sivakumar et al., 2019).

To enable the accessibility of proteins to their target sequences, the chromatin must be remodeled into a less compacted structure, whereas a more compacted structure is associated with transcriptional repression. Furthermore, the chromatin structure is highly dynamic, and its remodeling contributes to many functions in the cell (Felsenfeld and Groudine, 2003; Deng and Chang, 2007; Bassett et al., 2009; Pombo and Dillon, 2015).

To understand how the domains derived from the hierarchical organization of chromatin are formed, and how this organization is highly dynamic, it is necessary to visualize how DNA interacts with diverse proteins. At the first level of compaction, in an interphase chromosome, there exists a 6.5 nm diameter cylinder-like structure called nucleosome, which is formed by histone octamers with 146 base pairs (bp) of DNA wrapped around this core in 1.6 turns (Felsenfeld and Groudine, 2003; Bassett et al., 2009; McGinty and Tan, 2015; Pombo and Dillon, 2015).

This tetramer is formed by two heterodimers of the histones H3 and H4, which are flanked by two heterodimers of H2A and H2B histones in a structure known as the “histone core.” From this core, eight N-terminal and two C-terminal ends project out at defined locations. These are susceptible to a large number of post-translational modifications, some of which are recognized by protein complexes involved in the remodeling and maintenance of chromatin (McGinty and Tan, 2015).

Super-resolution nanoscopy (stochastic optical reconstruction microscopy [STORM]) revealed that the nucleosomes can organize into discrete groups called “nucleosome clutches” that lack an organized structure. The number of nucleosomes per clutch is variable; they are interspersed with nucleosome-depleted regions, and the nucleosome density is cell-type specific (Ricci et al., 2015).

## Chromatin Loops

**FIGURE 1:**
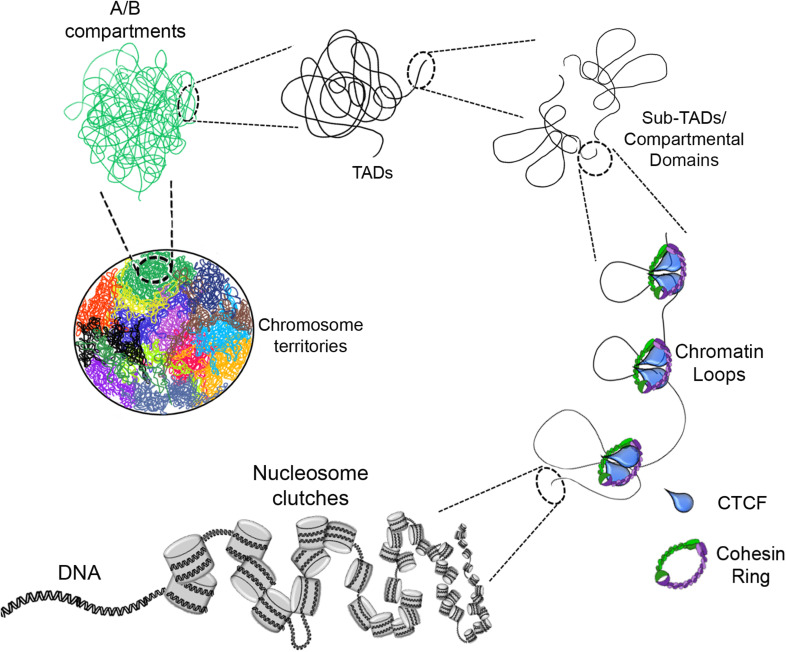
Eukaryotic chromatin organization. The DNA interacts with histone octamers and aggregates forming nucleosome clutches. In the next level of compaction are the chromatin loops which are formed by loop extrusion and in a greater extent stabilized by CTCF and the cohesin ring. Chromatin loops are the base of compartmental domains, sub-TADs and TADs which range from ten of kb to Mb structures with delimited boundaries and high-rate interactions inside of these domains. A/B compartments is the next level, where can be determined by gene content, epigenetic marks, DNase hypersensitivity and nuclear localization. Finally, there are the chromosome territories which are the localization of each chromosome inside the nucleus (each color represents a different chromosome).

The next level of compaction consists of the so called “chromatin loops.” These structures have an average size in the kilobase (kb) scale (Figure 1). They are important because they allow a finer regulation of the transcriptional process by enabling contacts between distant regulatory elements such as: enhancer – promoter, silencer – promoter or insulator – insulator (Fraser et al., 2015; Rowley and Corces, 2018). Changes in the contacts between these loops can drive differential gene regulation and consequently, gene expression (Greenwald et al., 2019).

In vertebrates, these chromatin loops are formed and stabilized through interactions with the architectural protein called CCCTC-binding factor (CTCF) and the cohesin complex. Analysis of Hi-C data has revealed that CTCF-binding motifs occur in a convergent orientation (forward-reverse), which serve as docking sites for CTCF to bind to DNA in a way that facilitates its positioning in a restricted 3D space (Fudenberg et al., 2016; Rowley and Corces, 2018).

Interestingly, CTCF positioning along the genome is independent of the presence of cohesin, but cohesin localization is dependent of CTCF. This shows that CTCF recruits and leads cohesin to the target loci (Wendt et al., 2008). This observation suggests a joint activity between cohesin and CTCF.

Later, *in silico* analyses (Rao et al., 2014) revealed that CTCF is involved in setting up the chromatin loops. CTCF has eleven zinc fingers and uses different combinations of them to bind to the DNA and to different proteins (Filippova et al., 1996). Recently, the N-terminal end of CTCF was demonstrated to be necessary for loop formation as it is involved in cohesin retention (Pugacheva et al., 2020), whereas its C-terminal is involved in CTCF dimerization (Pant et al., 2004; Hansen et al., 2019).

Moreover, the first two zinc fingers of CTCF and likely the 3D configuration of the CTCF/Cohesin/DNA complex appear to be involved in cohesin retention (Pugacheva et al., 2020). Accordingly, there are reports showing that depletion of cohesin, CTCF, or the cohesin-loader protein, NIPBL, causes disruption of the chromatin loop domains (Nora et al., 2012; Wutz et al., 2017), whereas depletion of WAPL (a cohesin release factor) causes reinforcement of the stability of the loops. This effect has also been observed at the topologically associating domain (TAD) boundaries (Haarhuis et al., 2017).

Currently, CTCF is recognized as the only protein essential for the formation of chromatin loops in mammals. A model, called the “loop extrusion model,” has been proposed for the formation of these loops, according to which, the cohesin complex, comprising the SMC proteins and RAD21, is directed to the chromatin with the help of NIPBL protein, and together “pull” the DNA strand until the cohesin ring gets stuck with CTCF, and thus, forms the loops (Fudenberg et al., 2016; Rowley and Corces, 2018).

Three hypotheses have been postulated to explain how the chromatin loops are formed; in the first, DNA extrusion is triggered by a diffusion gradient generated by cohesin itself (Brackley et al., 2017). The second hypothesis suggests that the cohesin complex, through ATP hydrolysis, functions as a motor that pulls the DNA strand (Terakawa et al., 2017; Vian et al., 2018), while the third hypothesis proposes that the extrusion is actually generated by RNApol II (Davidson et al., 2016; Ocampo-Hafalla et al., 2016; Stigler et al., 2016; Busslinger et al., 2017) suggesting that transcription of the nearby sites is really what defines the formation of these domains. There are experimental evidences that support the three hypotheses and they may not necessarily be mutually exclusive since the cohesin complex and the RNAPol II can work together promoting transcription and compartmental domains (Rowley et al., 2019).

## Topologically Associating Domains (TADs)

At the next level of compaction are the TADs (Figure 1). Through 5C and Hi-C experiments it was found that chromosomes are partitioned into domains that form regulatory landscapes and whose boundaries correspond to replication domains (Pope et al., 2014). Currently, such domains are known as TADs, and they generally have sizes in the mb scale (for e.g., TADs have an average size of ∼900 kb in mice but could be larger or smaller) (Dixon et al., 2012; Nora et al., 2012).

These structures are characterized by well-defined boundaries flanked by architectural proteins. Such delimitation results in strong interactions among the elements that are in the same TAD, but poor or null interaction between elements that are in different TADs (Pope et al., 2014; Huang et al., 2019; Beagan and Phillips-Cremins, 2020).

Computational analyses carried out using different algorithms such as “arrowhead” have revealed that multiple interactions occur between DNA sequences within the TADs, which are in close proximity in the 3D space, and enrichment of CTCF at those sites, including at the boundaries of these domains (Rao et al., 2014).

**FIGURE 2:**
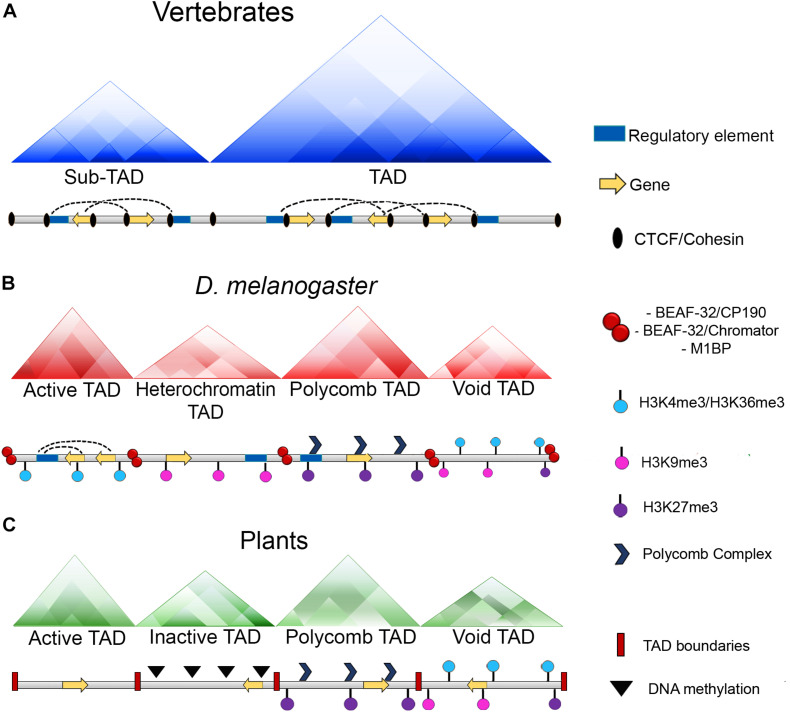
Chromatin organization among different organisms. **(A)** In vertebrates, chromatin loops are formed and stabilized by the presence of CTCF and the cohesin complex. Moreover, TAD boundaries present an enrichment of CTCF and the cohesin complex which act as insulators and keep these domains detached. **(B)** In *Drosophila*, chromatin loops exist, however, there is not an enrichment of the dCTCF ortholog with the cohesin complex at the boundaries of these domains, instead, this role it is carried out by other architectural proteins of the fly as BEAF-32, CP190, chromator or M1BP. Additionally, in *Drosophila* TADs can be classified according to their epigenetic states: Active TADs which possess an enrichment of active histone marks as H3K4me3 and H3K36me3; heterochromatin TADs which have an enrichment of repressive marks as H3K9me3; Polycomb TADs which are enriched with the presence of Polycomb complexes and the H3K27me3 mark and void TADs which do not have a defined landscape. **(C)** In plants, CTCF is not conserved and there is not a report of any protein with insulator activities. However, the existence of TAD-like domains has been reported and as well as *Drosophila*, these domains can be classified in four distinct categories which are: Active TADs; inactive TADs characterized by a high degree of DNA methylation; Polycomb TADs and void TADs.

Furthermore, high resolution Hi-C maps have revealed the existence of smaller domains that were named as sub-TADs or compartmental domains. These have an average size of 200 kb and are enriched with specific chromatin marks that are associated with transcriptional activation or with transcriptional repression (Figure 2) (Rao et al., 2014; Rowley et al., 2017).

Interestingly, TADs seem to have highly conserved features in mammals. A notable characteristic of almost all TADs is the presence of CTCF along with the SMC-cohesin complex at their boundaries, varying from 75 to 90% of all boundaries depending on the cell type (Dixon et al., 2012; Bonev et al., 2017). Moreover, CTCF sites located at these boundaries present a convergent orientation. It has been reported that a change in the orientation or the removal of a single CTCF site can shift the position of a TAD boundary or even completely abolish it (de Wit et al., 2015; Guo et al., 2015; Lupiáñez et al., 2015; Sanborn et al., 2015).

Thus, this indicates that TADs are also possibly formed by the loop extrusion mechanism, and many of them result from an equilibrium between the loading and release of cohesin along the chromatin (Nuebler et al., 2017). Furthermore, when cohesin is not loaded into chromatin and the TAD boundaries are affected, restoration of cohesin reverts this effect, indicating that this process is highly dynamic (Rao et al., 2017). In contrast, data indicates that although CTCF and cohesin are present in almost all TAD boundaries, cohesin depleted cells seem to have a randomized localization of these boundaries compared to wild-type cells, raising the possibility that TADs can be generated through spontaneous contacts in the chromatin and that other loop-extruding mechanisms may exist (Bintu et al., 2018).

As mentioned previously, some TAD boundaries are CTCF-independent, in that they are not affected by CTCF loss (Nora et al., 2012). In these cases, it has been suggested that the establishment of the TADs may be due to transcription (Dixon et al., 2012; Bonev et al., 2017). Supporting this, experimental data show that some TAD boundaries appear near promoters of recently transcribed genes during cell differentiation in a CTCF-independent manner (Bonev et al., 2017). Hence, these results support the hypothesis that some TADs are established by transcription *per se* (Rowley et al., 2017). However, transcription does not seem to be sufficient for the establishment of these boundaries or at least, in some of the cases.

In one study, treatment of K562 cells with RNAse A followed by Hi-C assays demonstrated that the lack of RNA did not disrupt TADs but had a mild effect disrupting the compartmental interactions. Additionally, inhibition of transcription affected TAD boundary strength since more interactions between TADs were observed, and TAD weakening was independent of CTCF. These results favor a model in which TAD formation occurs through DNA–protein and protein–protein interactions instead of RNA-based interactions (Barutcu et al., 2019).

Also, since transcription and RNA are inhibited or degraded, respectively, TAD weakening may occur due to loss of part of the nuclear pool of CTCF or cohesion complexes (Barutcu et al., 2019). These results agree with another study in that cohesin degradation resulted in the disappearance of TADs that then reappeared following rescue with cohesion even in the absence of transcription (Vian et al., 2018). Thus, the activities of the cohesin ring are important for TAD formation and maintenance.

On the other hand, in studies in early mouse embryos, transcriptional inhibition with α-amanitin did not prevent TAD formation, whereas replication abolishment with aphidicolin had a negative effect on TAD formation (Du et al., 2017; Ke et al., 2017). This suggests a potential role for replication in TAD establishment, at least during early embryonic development.

## Chromatin Compartments

Recent advances in the study of the organization of chromatin using Hi-C or chromatin interaction analysis by paired-end tag sequencing (ChIA-PET) have shown a higher level of compaction known as “chromatin compartments.” These mega-structures are classified as compartment A for open chromatin state or compartment B for closed chromatin state (Figure 1), depending on whether the chromatin structure in these regions is loose or compacted (Capelson and Hetzer, 2009; Lieberman-Aiden et al., 2009; Fraser et al., 2015; Rowley and Corces, 2018).

Type A compartments are characterized by a high content of transcriptionally active genes and correlate with active histone marks including H3K9ac and H3K27ac, high GC content, as well as hypersensitivity to DNAse I. Thus, A compartments have permissive transcriptional environment, although it should be noted that genes that are silenced may also exist to a lesser extent within these regions (Guelen et al., 2008; Giorgetti et al., 2016).

On the other hand, type B compartments are characterized by the opposite features, including a high content of silenced genes and correlate with repressive histone marks such as H3K9me2, H3K9me3, and H3K27me3, poor or null DNAse I hypersensitivity, and late replication timing. Further, as in the case of type A compartments, the type B compartments may also contain exceptions in terms of genes that are transcriptionally active (Guelen et al., 2008; van Steensel and Belmont, 2017).

The localization of the chromatin compartments is non-random in the nucleus, and this preferential distribution is highly correlated with its intrinsic characteristics. Hi-C data have shown that A compartments are located preferentially in the central region of the nucleus as well as in adjacent regions close to the nuclear pore complexes (NPCs) (Solovei et al., 2009; Ou et al., 2017; Stevens et al., 2017; Buchwalter et al., 2019).

B compartments are preferentially located at the periphery of the nucleus, interacting with elements of the nuclear lamina, which constitutes a predominantly repressive environment. These results are supported by electron microscopic studies that have shown heterochromatin to be preferentially located and clustered near the nuclear lamina in most cell types (Capelson and Hetzer, 2009; Ou et al., 2017; Stevens et al., 2017; Buchwalter et al., 2019).

It is important to mention that this array of compartments generally occurs in almost all cell types, but there are some exceptions where B compartments may be found located inside the nucleus and A compartments located adjacent to or interacting with the nuclear lamina (Solovei et al., 2009). It is, however, important to keep in mind that this distribution is not a coincidence and is highly correlated with the cell function (Rego et al., 2008; Shevelyov et al., 2009; Peric-Hupkes et al., 2010; Buchwalter et al., 2019).

The regions where B compartments interact with the components of the nuclear lamina are known as “lamina associated domains” (LADs). It has been reported that in mammals approximately 10% of the total genes are located in these domains, whereas up to one third of the whole genome are represented in these domains (Peric-Hupkes et al., 2010; Kind et al., 2015).

## Chromosome Territories

The last level of compaction, known till date, is referred to as “chromosomal territories” (CTs) (Figure 1). The first experimental data and visuals of these mega-structures were obtained through fluorescent *in situ* hybridization (FISH) techniques where each chromosome can be labeled with a different fluorescent probe for individual detection of each chromosome (Cremer et al., 1984; Fawcett et al., 1994).

Further refined methods such as 3D FISH in combination with light optical serial sectioning of nuclei by laser confocal microscopy and 3D image reconstruction, allowed for the determination of the spatial arrangement of CTs and their substructures (Cremer et al., 2008; Cremer and Cremer, 2010). Because of these new techniques, it was determined that the distribution of CTs into the nucleus is non-random (Cremer et al., 2001, 2003; Bolzer et al., 2005).

Chromosomal territories refer to the position of each chromosome in the nucleus. Experimental data have revealed that, globally, the sequences contained in each chromosome tend to interact with sequences located in the same chromosome, and at the same time tend to be excluded from sequences in other chromosomes. Thus, in this way, chromosomes are restricted to specific loci instead of being scattered across the nucleus (Cremer and Cremer, 2010; Sarnataro et al., 2017; Fritz et al., 2019).

Currently, it is well known that chromosomes possess variable gene content among them, and previous studies have shown that chromosomes with higher gene density tend to be located at the interior of the nuclei, whereas chromosomes with a poor gene content are preferably located in the nuclear periphery (Cremer et al., 2001, 2003; Bolzer et al., 2005).

Interestingly, CTs have been shown to be susceptible to relocalization across the nucleus depending on the differentiation state of some cell types. During cellular differentiation of murine cerebellar Purkinje neurons, CTs change their positions at the end of the fifth day post-partum (Martou and De Boni, 2000). Whereas, in rod cells of nocturnal mammals, the CTs begin to reposition after the sixth day post-partum, resulting in all euchromatin being shifted to the nuclear periphery and the heterochromatin to the center of the nucleus (Solovei et al., 2009).

Interchromosomal contacts between CTs are approximately three orders of magnitude weaker than intrachromosomal contacts (Lieberman-Aiden et al., 2009; Rao et al., 2014). Intrachromosomal contacts are favored and enriched between domains that are rich in highly expressed genes (Sarnataro et al., 2017). However, these interactions do not occur randomly, which suggests they are important for the activation and regulation of genes encompassed in these loci.

## Is the Nuclear Architecture the Same in All Eukaryotic Organisms?

As detailed earlier, the chromatin architecture is intrinsically linked with cellular and developmental patterns. This begs the question whether the nuclear architecture is different between the eukaryotes given the variations in genome sizes (Oliver et al., 2007; Pellicer et al., 2018), and different chromosome and gene numbers in different organisms (Hardison, 2003; Touchman, 2010).

In principle, at a very basic level of the chromatin (at the histone level), there seem to be no major differences in their composition, and most of the canonical and variant histones are highly conserved (discussed below). However, the first clear difference in the genome architecture between eukaryotes is found at the level of the chromatin loops. As discussed earlier, in vertebrates, these structures are formed by interaction and stabilization between CTCF and the cohesin complex (Pant et al., 2004; Wendt et al., 2008; Rao et al., 2014; Fudenberg et al., 2016; Rowley and Corces, 2018; Hansen et al., 2019; Pugacheva et al., 2020).

CTCF is a highly conserved protein during evolution and is present in almost all bilaterian metazoans (with a few exceptions like *Caenorhabditis elegans*) (Heger et al., 2012). Over 93% of amino acids is reportedly identical between the human and chicken CTCFs (Filippova et al., 1996). The 11 zinc fingers constitute the ultra-conserved region of CTCFs, which is identical from *Drosophila* to humans, suggesting conserved functions for this domain of the protein (Moon et al., 2005; Bartkuhn et al., 2009; Bushey et al., 2009; Cuddapah et al., 2009). The N- and C-terminal ends present more variation between organisms, although recent reports indicate that both these domains are necessary for cohesin recruitment and stabilization, at least in mammals (Pugacheva et al., 2020).

Interestingly in invertebrates, like in *Drosophila*, dCTCF is not essential for the establishment of chromatin loops (van Bortle and Corces, 2012; Ong and Corces, 2014). This may be explained in part by the presence of cohesin complexes independent of dCTCF in genes that are transcriptionally active (Misulovin et al., 2008), suggesting that the ortholog in *Drosophila* does not contain the cohesin-interaction domain. Moreover, it has been demonstrated that BORIS, a germ-cell specific CTCF paralog in mice, which differs from CTCF in its N- and C-terminal ends, is not capable of anchoring cohesin to the chromatin (Pugacheva et al., 2020), highlighting the importance of these domains in cohesin interaction.

In flies, dCTCF has been found at tens of thousands of independent sites throughout the genome (Bushey et al., 2009; Cuddapah et al., 2009), and the distribution pattern suggests that this protein may play a role both in the individual regulation of genes, as well as in the global organization of the genome. However, dCTCF co-localizes to the boundaries of many domains with other architectural proteins that are exclusive to the fly, such as CP190, BEAF-32, and Mod (mdg4) (Figure 2) (Bartkuhn et al., 2009; van Bortle and Corces, 2012).

These data suggest that although dCTCF cannot recruit the cohesin complex for the formation of chromatin loops, it is possible that this protein binds to other architectural proteins that are exclusive to the fly and thus, delimits the formation of different domains in the genome (van Bortle and Corces, 2012). Furthermore, genetic and biochemical evidence demonstrates that some of these proteins act in complexes, and are distributed along the genome in different combinations, which provides specificity in the regulation of gene expression (Gerasimova et al., 1995; Melnikova et al., 2004, 2017, 2019; Soshnev et al., 2013; Vogelmann et al., 2014; Glenn and Geyer, 2019; Kirchanova et al., 2019).

Another interesting question that arises from these data regarding the architecture of the genome is how are TADs established in invertebrates? In *Drosophila*, Hi-C experiments demonstrated the existence of discrete domains with many interactions in the chromosomes that can be classified as TADs according its epigenetic states. Thus, *Drosophila* TADs can be partitioned into four classes of TADs which are known as active TADs, heterochromatin TADs, Polycomb TADs, and void TADs (Figure 2) (Sexton et al., 2012; Szabo et al., 2018). These domains are smaller than those in mammals, with an average size of ∼100 kb (Hou et al., 2012; Sexton et al., 2012). However, high resolution Hi-C analyses revealed smaller domains that are contiguously partitioned along the genome with sizes ranging between 3 and 460 kb (Wang et al., 2018).

An interesting characteristic of TADs in *Drosophila* is that dCTCF or cohesin are not significantly enriched at the TAD boundaries as in the mammals (Ulianov et al., 2016; Cubeñas-Potts et al., 2017). Instead, they are enriched with pairs of architectural proteins, such as BEAF-32/CP190, BEAF-32/chromator or M1BP (Figure 2) (Hou et al., 2012; Sexton et al., 2012; Ulianov et al., 2016; Hug et al., 2017; Ramírez et al., 2018; Wang et al., 2018). Moreover, RNApol II and transcription factors are also found to be enriched at the borders of TADs (Bushey et al., 2009).

Despite its high level of evolutionary conservation in metazoans, CTCF is not present in *C. elegans* (Heger et al., 2012). This suggests that TADs do not exist in this organism. However, a study found that in *C. elegans* the X chromosome with dosage compensation contains structures approximately 1 Mb in size that contain multiple self-interacting domains resembling TADs. These chromosomes also contain a condensing complex known as the dosage compensation complex (DCC), which is located at the boundaries of these domains. Besides, it was observed that these domains diminished or lost strength in DCC mutants, providing insights into how DCCs reshape the topology of the X chromosome and their implications in gene expression in *C. elegans* (Crane et al., 2015).

Finally, it is well known that CTCF is absent in plants (Heger et al., 2012), although the existence of TAD-like domains has been reported previously in plants as tomato, sorghum, rice or maize. The characteristics of these TAD-like domains consist in an enrichment of *cis* interactions within domains and regions of open chromatin, active histone marks and the absence of DNA methylation and transposable elements (Dong et al., 2017).

Interestingly, as well as in *Drosophila*, (Sexton et al., 2012) plant TAD-like domains can be partitioned into four types of domains which are repressive domains (associated with DNA methylation), open chromatin (active domains), Polycomb domains (enriched with H3K27me3 mark) and intermediate domains which lack distinctive features (Figure 2) (Dong et al., 2017).

In the case of Polycomb domains, they show changes in the levels of the H3K27me3 mark at the domain borders, also, repressive domains are depleted of epigenetic features at the domain borders, suggesting that chromatin states, epigenomic features and active transcription may play an important role in forming the chromatin domain boundaries. Moreover, similar to what happens in *Drosophila*, it has been reported the existence of compartmental domains (Dong et al., 2017).

Eigenvector analysis of Hi-C data, found that compartments that can be globally classified as A or B at the same time, have high levels of H3K27me3 mark allowing its grouping into TE-rich or H3K27me3 rich regions, indicating that Polycomb proteins could be involved in local chromatin organization (Dong et al., 2017). Two important features in plants are described, on one hand, the lack of a CTCF homolog (Heger et al., 2012) and on the other hand, the lack of synteny between a specific chromatin domain between plant species. This can be compared against mammalian TAD conservation and its relationship with CTCF binding, therefore, it has been proposed that in plants other factors could mediate the establishment of these domains (Dong et al., 2017).

Currently, all available data indicate that in vertebrates CTCF is a universal factor that plays a fundamental role in chromatin loops and TADs establishment. Nevertheless, in the case of *Drosophila*, despite of the existence of the ortholog dCTCF, this protein does not play an essential role for the establishment of chromatin loops and TADs, and this activity relies on other architectural proteins specific of *Drosophila*.

As discussed before, one of the reasons are the differences of the N and C terminal ends between CTCF and dCTCF which are important for cohesin retention. Recent reports have shown that ISWI CRCs contribute to the binding of CTCF at its target sites (discussed further below). These data arise the question if in organisms like *Drosophila* or plants, where the orthologs of CTCF do not seem to have an essential role at TAD boundaries or where CTCF is totally absent, CRCs play an important role in directing architectural proteins to their target sites in order to control chromatin looping and TAD formation.

## Modifications in the Chromatin Structure

Up to this point, far from being a static entity, the chromatin structure is highly dynamic, varying between euchromatin or heterochromatin states to allow for transcription of specific regions of the genome. However, it is important to mention that all the known ways in which chromatin can be remodeled occur at the nucleosome level, and only three mechanisms have been described.

First, histone post-translational modifications (HPTMs) generally occur at the N-terminal ends of histones. They are the result of the activity of specialized groups of enzymes, such as histone acetyltransferases, which are involved in the acetylation of certain lysine residues (Marmorstein and Zhou, 2014), and histone methyltransferases, which are involved in methylation, whereas phosphorylation is mediated by different kinases (Iizuka and Smith, 2003).

Among various HPTMs, acetylation on K9 and K14 of histone H3, as well as H4K5S is correlated with a transcriptionally active state, whereas deacetylation of these residues is involved in silencing of transcription (Fischle et al., 2003; Li et al., 2007). Similarly, phosphorylation on S10 and S28 of histone H3 is correlated with activation of transcription, whereas H3K9P phosphorylation triggers chromatin condensation and subsequent transcriptional silencing (Fischle et al., 2003).

Multiple modifications can also occur on the same residue. Di and trimethylation have an important role in some physiological processes; H3K4me2 marks genes that are both transcriptionally active and silenced, whereas H3K4me3 is only found in genes that are transcriptionally active (Santos-Rosa et al., 2002).

Another type of HPTM includes ubiquitylation. H2AK119ub is reported to have a role in transcriptional repression because of its role in the repression of a subset of chemokine genes (Zhou et al., 2008), Polycomb silencing (Wang et al., 2004), and X chromosome inactivation (Fang et al., 2004). On the other hand, H2AK13ub and H2AK15ub are involved in the signaling of the double-strand break repair pathway (Mattiroli et al., 2012; Fradet-Turcotte et al., 2013).

Crotonylation is another HPTM that has been reported across species from yeast to humans (Tan et al., 2011). H3K9cr is associated with transcriptional activation (Andrews et al., 2016). Recently, H3Q5 serotonylation was reported to promote the recruitment of TFIID together with the H3K4me3 mark, suggesting its role in transcriptional activation (Farrelli et al., 2019).

Further, diverse types of HPTMs have also been described in the histone globular domains including methylation, acetylation, or ubiquitylation of K residues; methylation of R residues; or phosphorylation of S residues that contribute to remodeling of chromatin and have a role in the regulation of gene expression (Suganuma and Workman, 2011). The functional groups present in some residues also serve as recognition and anchor sites for various elements, such as the chromatin remodelers (discussed below) that bind and carry out their functions at these sites (Cairns, 2009; Alekseyenko et al., 2014). Furthermore, these functional groups are also recognized by various proteins that function as gene co-activators or co-repressors. For instance, HP1, which is involved in the maintenance and formation of heterochromatin, recognizes the histone mark, H3K9me3 (Canzio et al., 2011).

All the specific modifications on histones have relevant biological implications at different organizational levels. They can direct different activities in different regions and regulatory elements. Through regulatory elements such as enhancers, they can influence groups of genes at a domain level. They may also have an effect at the chromosomal level, as in the case of the silencing of the X chromosome (Jenuwein and Allis, 2001; Fischle et al., 2003).

Second, remodeling may be mediated by histone variants. These are histones that differ from the canonical histones in several aspects. For example, canonical histones are deposited during the S phase of the cycle, whereas deposition of the variants can occur at different stages. Additionally, the variants often have different amino acid residues, extra domains, or lack some domains compared to the canonical histones. The nucleosomes that contain these variants also have different properties. They tend to be either more labile or more stable. Further, the presence of some variants in the nucleosome indicate regions where DNA damage has occurred. Thus, the presence of these variants in the nucleosome can trigger a specialized function (Ahmad and Henikoff, 2002; Felsenfeld and Groudine, 2003; Iizuka and Smith, 2003; Schneiderman et al., 2012).

For example, the histone variant H3.3 is deposited at telomeric regions by a complex composed by the chaperone DAXX and the ATPase subunit ATRX which is a CRC member of the SWI/SNF family. H3.3 histone deposition specifically at telomeres by DAXX/ATRX complex of pluripotent and non-pluripotent cells has been proposed as a mechanism to facilitate the access to chromatin (Drané et al., 2010; Goldberg et al., 2010; Lewis et al., 2010). Furthermore, H3.3 variant has also been linked with repressive activities (Elsässer et al., 2015; Sadic et al., 2015).

Finally, the ATPase activity of CRCs can evict, slide, remove or deposit nucleosomes or histones, and are involved in the regulation of transcriptional activation by modifying the chromatin architecture at different regions. CRCs allow for regulation of transcription through the activation or repression of genes that control alternating euchromatin and heterochromatin states, which in turn, allows for regulation in gene expression (Saha et al., 2006; Cairns, 2009). It is important to mention that, in this review, we will focus specifically on chromatin remodelers, and their impact on the genome architecture.

## Chromatin Remodeling Complexes

Chromatin remodeling complexes (CRCs) can be described as specialized multiprotein machineries that allow access to DNA by temporarily modifying the structure or composition of nucleosomes (Cairns, 2009). These complexes use the energy from ATP to restructure, mobilize, and expel nucleosomes to regulate the access to DNA (Owen-Hughes, 2003). Most chromatin remodelers form large complexes composed of multiple accessory subunits and a central core that contains the ATPase catalytic activity. The accessory subunits generally contain interaction domains that regulate the enzymatic activity of the complex, facilitating the binding of transcription factors and other chromatin-modifying enzymes, and thus guide the complex to the modified DNA and/or histones (Hargreaves and Crabtree, 2011).

**FIGURE 3:**
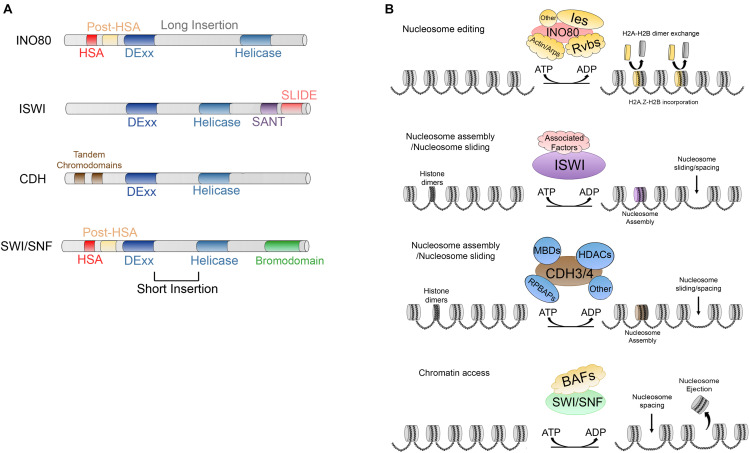
**(A)** Main characteristics and domains of INO80, IWSI, CHD, and SWI/SNF families. **(B)** ATP dependent activities carried out by; INO80, which is responsible of nucleosome editing by exchanging H2A-H2B and H2A.Z-H2B dimers; ISWI and CDH are involved in nucleosome maturation and nucleosome assembly; additionally, ISWI and CDH members are involved in nucleosome spacing and sliding; SWI/SNF members trigger chromatin access through nucleosome spacing, nucleosome ejection as well as dimer eviction.

Because of the large number of genetic interactions and the difficulty in characterizing them biochemically, CRCs have been classified based on the degree of conservation of the helicase/ATPase subunit and by the unique flanking domains that confer different functions (Saha et al., 2006). To date, four CRCs families have been described in eukaryotes and all of them are involved in several biological processes (Figure 3A). Among these, we will highlight the activation and regulation of the RNApol II (Hirschhorn et al., 1992; Armstrong et al., 2002), silencing and transcriptional repression (Wade et al., 1999), histone exchange (Jenuwein and Allis, 2001; Drané et al., 2010; Goldberg et al., 2010), and DNA repair and homologous recombination (Saha et al., 2006; Clapier and Cairns, 2009; Juhász et al., 2018; Lovejoy et al., 2020).

The INO80 (Inositol requiring 80) complex is a member of the CRC family and was initially purified and characterized in *S. cerevisiae*. It is made up of 15 principal subunits, which are as follows; INO80 (ATPase domain), Rvb1, Rvb2, Arp4 (actin related protein-4), Arp5, Arp8, actin, Nhp10 (non-histone protein 10), Anc1/Taf14, Ies1 (Ino eighty subunit-1), Ies2, Ies3, Ies4, Ies5, Ies6 (Shen et al., 2000, 2003).

INO80 complex is highly conserved in evolution and the human ortholog, hINO80, contains almost all the subunits excepting Hhp10, Anc1/Taf14 and Ies3-5, but possess five unique subunits (Jin et al., 2005). Also, it is conserved in *Drosophila*, where it has 19 subunits (Prozillo et al., 2019). The main characteristic of the members of this family of CRCs is a split DExx/Helicase domain separated by a long insertion. Additionally, they possess an HSA domain and post-HSA domains at their N-terminal ends (Figure 3A) (Saha et al., 2006; Clapier and Cairns, 2009). This CRC is involved in DNA transcription, DNA repair, and homologous recombination (Downs et al., 2004; Morrison et al., 2004; van Attikum et al., 2004; Tsukuda et al., 2005; Saha et al., 2006; Clapier and Cairns, 2009; Kusch et al., 2014).

INO80 CRCs members participate in nucleosome editing (Figure 3B) which is the replication-independent removal of histones and replaces them either with canonical or histone variants within the nucleosomes (reviewed by Clapier et al., 2017). These activities were described in the yeast complex SRW1C, the *Drosophila* Tip60 complex, mammalian P400 and snf2-related CBP activator protein (SRCAP) (Kusch et al., 2004; Mizuguchi et al., 2004; Ruhl et al., 2006; Goldberg et al., 2010).

INO80 translocates along the DNA and promotes the exchange of H2A.Z-H2B dimer more efficiently than H2A-H2B containing dimers (Brahma et al., 2017). Cryo-EM and single-particle reconstruction techniques have determined the core of the INO80 complex at a resolution of 3.7Å. The conserved core has a “ratchet-like” mechanism of action, where the INO80 subunit first unwraps the nucleosome DNA entry and grips histones in joint with Arp5 and Ies6 subunits. Later, through multiple steps of sliding triggered by ATP-dependent pumping, Arp5-Ies6 holds the DNA and through motor force, generates a transient DNA loop which likely exposes the H2A-H2B histone dimer for nucleosome editing (Eustermann et al., 2018).

ISWI (imitation switch) members were initially identified for their nucleosome remodeling activities in *Drosophila* embryo extracts through *in vitro* assays (Tsukiyama et al., 1995; Ito et al., 1997; Varga-Weisz et al., 1997). The members of this family are characterized by a DExx/Helicase domain split by a short insertion and a SANT (named after switching-defective protein 3 [Swi3], adaptor 2 [Ada2], nuclear receptor co-repressor [N-CoR] and transcription factor [TFIIIB]) domain followed by a SLIDE domain at their C-terminal end (Aasland et al., 1996; Boyer et al., 2002). Together, these domains form a module that can recognize DNA and unmodified histone tails (Boyer et al., 2004).

ISWI members are diverse and may contain other domains that confer specificity, such as DNA binding/histone fold domains, PHD (plant homeo-domain), bromodomains or additional DNA binding motifs (Langst et al., 1999; Hassan et al., 2002; Carey et al., 2006; Ferreira et al., 2007). ISWI is part of several CRCs in different organisms. Originally, three ISWI-dependent complexes were characterized and purified from embryo extracts in *Drosophila* which were named dNURF (Tsukiyama et al., 1995), dCHRAC (Varga-Weisz et al., 1997) and dACF (Ito et al., 1997). In mammals at least six ISWI-dependent CRCs have been described, WICH, NORC, NURF, ACF, RSF and CHRAC (Erdel and Rippe, 2011; Aydin et al., 2014), each complex contains one of two conserved ATPase subunits SMARCA5 (also known as SNF2H) or SMARCA1 (SNF2L) associated with one or more accessory subunits (Barak et al., 2003; Banting et al., 2005; Aydin et al., 2014).

ISWI members act facilitating nucleosome sliding of histone octamers and promotes histone maturation (Figure 3B) (Längst and Becker, 2001; Clapier et al., 2017). Binding of human ISWI SNF2H induces histone deformation which is important for its catalytic activity (Sinha et al., 2017) recently, Cryo-EM studies have shown that the ISWI complex of *Saccharomyces cerevisiae* also triggers DNA distortion and translocation after ISWI activation, showing an unperturbed histone core structure with the exception of the H4 tails, this mechanism is identical to the human SNF2H mechanism, suggesting a common DNA translocation mechanism (Yan et al., 2019). ISWI members are associated with diverse biological processes. They may participate in maintaining correct spacing between nucleosomes, thus assisting in RNApol II activation. Moreover, it has been reported that they can also act on nucleosomes that are not acetylated in regions that are not transcriptionally active (Langst et al., 1999; Corona et al., 2002; Clapier and Cairns, 2009).

The CHD (chromodomain-helicase-DNA binding) family, was originally identified in *X. laevis.* This family of CRCs is also conserved from yeast to humans (Marfella and Imbalzano, 2007). CDH members has a DExx/helicase domain (known as CHD/NuRD) split by a short insertion. The unique characteristic of the members of this family is the presence of two tandem chromodomains (Figure 3A) alternating with diverse DNA binding domains such as SANT, CR1-3, PHD or BRK (Clapier and Cairns, 2009) at their N-terminal end. CHD CRC members act by sliding the nucleosomes to facilitate the activation of transcription. Moreover, they are involved in diverse processes including elongation of transcription although they can promote nucleosome maturation (Figure 3B) (reviewed by Clapier et al., 2017). On the other hand, some other members, such as Mi-2/NuRD found in humans, may have repressive roles due to their deacetylase activity and thus act as a CRC and a histone deacetylase (Denslow and Wade, 2007; Clapier and Cairns, 2009).

Finally, the SWI/SNF (switch defective/sucrose non-fermenting) proteins, which were originally described in *S. cerevisiae*, contain between 8 and 14 subunits. This family is characterized by a DExx/helicase domain separated in two by a short insertion. Further, the members contain a helicase-SANT domain (HSA) and a post-HSA near the catalytic domain and a bromodomain (which can bind acetylated residues of histones) at the C-terminal end (Figure 3A) (Denslow and Wade, 2007). Currently, there have been described various conserved subclasses (i) SWI/SNF and RSC in yeast (Peterson et al., 1994; Cairns et al., 1996), (ii) BAP (Brahma associated proteins) and pBAP in *Drosophila* (Mohrmann et al., 2004), and (iii) BAF (BRG1/BRM-associated factor) and pBAF (Polybromo-associated BAF complex) in mammals (Mohrmann and Verrijzer, 2005; Gangaraju and Bartholomew, 2007).

The members of this family trigger chromatin access through sliding and ejecting nucleosomes (Figure 3B) (reviewed by Clapier et al., 2017) and are involved in the activation of transcription, histone exchange, homologous recombination, and DNA repair (Whitehouse et al., 1999; Saha et al., 2006; Clapier and Cairns, 2009; Juhász et al., 2018; Lovejoy et al., 2020). However, the exact mechanisms of how these two processes are regulated are still unknown. On one hand, it has been reported that three domains, the Arp-7 and Arp-9 heterodimer, the helicase/SANT-associated (HSA) and the post-HSA and protrusion 1 act as regulators of DNA translocation which is a necessary activity for nucleosome sliding (Clapier et al., 2016). On the other hand, referring to nucleosome ejection, two non-mutually exclusive models have been proposed. In the first, DNA translocation could trigger the disruption of multiple DNA-histone contacts and possibly the H2A-H2B dimer might susceptible to ejection (Lorch et al., 2006; Yang et al., 2007; Clapier et al., 2017), whereas in the second mechanism the nucleosome adjacent to the one bound to the remodeler is the one that is ejected due to the processive DNA translocation that draws the linker DNA to the nucleosome bound and when this DNA is exhausted, the remodeler spools the DNA to the adjacent nucleosome ejecting the octamer (Clapier et al., 2017).

Genetic studies of these complexes have revealed activities in which they function cooperatively. Recently, another classification has been proposed based on the ATPase subunit position of the complex within the genome. Data from ChIP-seq experiments (Chromatin immunoprecipitation followed by high-throughput sequencing) of the eight catalytic subunits were compared to other epigenetic marks such as DNA methylation, histone modifications, nucleosome positioning, and chromatin contacts revealed by Hi-C experiments in the prostate cancer cell line LNCaP. A classification was proposed in which the chromatin remodelers are clustered into two functional groups (Giles et al., 2019).

Group 1 contains chromatin remodelers that are mainly (but not exclusively) associated with actively marked chromatin. This group contains SMARCA4, SNF2H, CHD3, and CHD4. Group 2 containing BRM, INO80, SNF2L, and CHD1 is mainly associated to repressed chromatin. Interestingly, both group 1 and 2 chromatin remodelers occupy sites within the TAD boundaries, intra-TADs, and around CTCF-binding sites. However, only group 1 remodelers are significantly enriched at active enhancer, promoter loop anchors, and even at long range chromatin loops. On the contrary, regions associated with LADs do not seem to require these chromatin remodeling activities as neither of the groups presented an enrichment at these sites. However, the latter does not exclude the possibility that other ATPases that were excluded from this study may be associated with or enriched at the LADs (Giles et al., 2019).

Consistent with this classification, through remodeler-nucleosome interaction assay (using MNase digestion to define nucleosomes, followed by remodelers ChIP-seq in embryonic stem cells, a study revealed that various remodelers such as SMARCA4, EP400, CHD1, CHD4, CHD6, and CHD8 occupied the same genomic regions, with most of them correlating with components of the basal transcriptional machinery, such as Pol II and TBP (TATA binding protein) at the transcription start sites (TSS) (Dieuleveult et al., 2016). Interestingly, this study reported that these remodelers worked together, with some of them functioning as activating remodelers for one class of genes and some of them counteracting the functions of these activating remodelers. In addition, the activating remodelers for one class of genes can act as inhibitor remodelers for other class of genes. Thus, remodelers can work together at regions adjacent to the promoter to elicit appropriate control of the gene. These data suggest that chromatin remodelers are complexes that can cooperate with each other to fulfill specific functions at various chromatin sites and are needed to maintain higher order chromatin structures.

## Remodeling Activities Required for Higher Order Chromatin Structure

### Promoter Clearance and CRC

**FIGURE 4:**
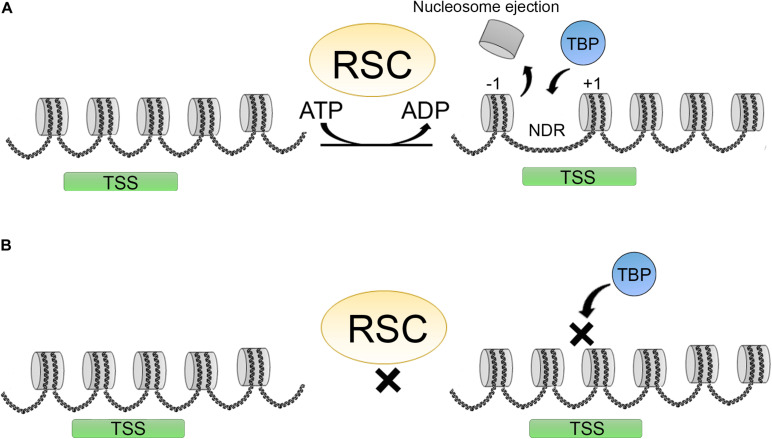
Promoter clearence carried out by the RSC complex. **(A)** RSC complexes act creating a nucleosome depleted region (NDR) around the TSS shifting the +1 nucleosome position, making this regions more accesible for different factors as TBP (TATA binding protein) thereby promoting transcription initiation. **(B)** Upon RSC depletion, NDRs around TSS are not formed and this impairs TBP binding and transcription initiation.

Several factors are involved in regulating the access to DNA: DNA base composition, HPTMs, presence of histone variants in the nucleosomes, histone chaperones, chromatin remodelers, and transcription factors. Transcription factors are implicated in recruiting chromatin remodelers and HPTMs to modulate their activities. RSC (remodeler of structure of chromatin) is a member of the SWI/SNF family. RSC participates in promoter clearance of a nucleosome-depleted region (NDR) by shifting +1 nucleosomes in the direction of the open reading frame (ORF), making the promoter more accessible (Figure 4A) (Lorch and Kornberg, 2017).

Other chromatin remodelers are also important for this function including ISWI and INO80, and some CHD’s such as CHD1 (Lusser et al., 2005). In humans, the RSC counterpart is PBAF. In yeast and human cells, the RSCs are considered to be the major remodeling complexes for transcription. RSC activity is also important in human cells for the choice of TSS. Depletion of RSC and other general transcription factors affects TBP binding and the +1-nucleosome positioning, affecting transcription initiation of a subset of genes (Figure 4B) (Kubik et al., 2018; Klein-Brill et al., 2019). Furthermore, it has also been established that RSC complexes can interact with +1 and −1 “partially wrapped” nucleosomes at the NDR or a subset of promoters, and promote their remodeling (Brahma and Henikoff, 2019; Schlichter et al., 2020).

Recently, the structure of RSC bound to a nucleosome has been resolved using CRYO-EM, in these studies it was found that RSC contacts not only the “partially wrapped” nucleosomes at the NDR, but also establishes contacts with the DNA promoter elements. RSC is organized into five main lobes, each with different functions. Through the main lobes it contacts the acetylated core histones, while the lobe that includes the ATPase contacts the promoter sequence, where the translocase activity of the complex takes place (Patel et al., 2019; Wagner et al., 2020). The ability of the CRCs to translocate along the DNA induces a superhelical torsion that is presumably used by other transcription factors or enzymes for different outcomes (Narlikar et al., 2013). Nevertheless, the translocation activities of the CRCs may impact higher order chromatin structures, as will be discussed in the subsequent sections.

INO80 complexes also have clear role in transcription. The Tip60 complex of *Drosophila* is involved in acetylating canonical H2A-H2B dimers, it promotes the exchange of these dimers at the body of certain stress response genes and, aids in RNA Pol II promoter release and elongation (Kusch et al., 2014). Importantly, studies in flies have demonstrated that some subunits such as YETI [which is part of the Bucentaur (BCNT) protein family] have an important role in nucleosome maintenance. *Tip60* and *Yeti* mutants display aberrant H2A and H2Av incorporation into chromatin, furthermore, other chromatin proteins and CRC are also affected, such as ISWI and HP1a, which in turn affects higher order chromatin structure (Messina et al., 2014). The effect of these mutants occurs not only at the protein level but also at the transcriptional level, since mRNA analysis of some of the chromatin binding proteins was also affected (Messina et al., 2014). All these data also provide a picture of the cooperation between these CRCs for the maintenance of higher order chromatin structure as will be discussed further below.

### CRC Association to Architectural Proteins

As discussed before, in vertebrates, CTCF is one of the main factors involved in the higher order chromatin structure. It functions by establishing different contacts either with DNA or with other proteins. Thus, it is not surprising that chromatin remodeling activities have been identified to be associated with this important protein. An interesting feature of the CTCF-binding sites, in vertebrates, is that this protein arranges close to 20 nucleosomes around its DNA binding site, and any disruption of this nucleosome array impedes CTCF-binding (Fu et al., 2008).

In a two-hybrid assay using the zinc fingers of CTCF as a bait, the carboxy domain of the SNF2 type ATPase, CHD8, was captured. CHD8 has two amino-terminal chromodomains and an SNF2 type domain. Reporter assays in cells with CHD8 knockdown revealed that the enhancer blocking activity was affected. H19 differentially methylated region (DMR) was used as the reporter construct. CTCF is important in directing CHD8 to the insulator site, and knockdown of CHD8 affected the insulator activity, but not CTCF binding. Moreover, CpG methylation of the adjacent loci was affected in the CHD8 knockdowns, and various sites were hypermethylated. Further, a reduction in the acetylation state of the nucleosomes around the insulator, but not at the CTCF-binding site was observed. The data revealed that CHD8-CTCF complex functions in altering the methylation state and histone modification in the vicinity of the insulator site (Ishihara et al., 2006).

**FIGURE 5:**
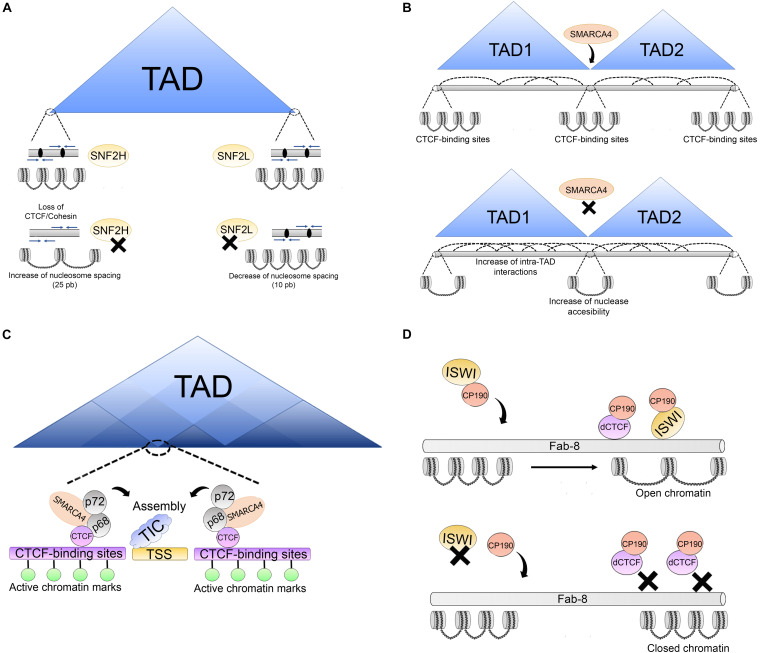
Association of architectural proteins with CRCs in vertebrates. **(A)** SNF2H and SNF2L are involved in the regulation of nucleosomes at TAD boundaries which are enriched with convergent CTCF binding motifs (blue arrows), CTCF binds and retains the cohesion complex (black ovals). After SNF2H depletion, nucleosome spacing increases over 25 bp promoting loss of CTCF/cohesin complexes. SNF2L depletion alters nucleosome organization of nucleosomes surrounding the CTCF sites and decreases nucleosome spacing over 10 bp. **(B)** SMARCA4 is a regulator of higher order chromatin structure. Upon SMARCA4 knockdown, weakening of TAD boundaries is triggered increasing intra-TAD and inter-TAD interactions. Additionally, this promotes changes in nucleosome positioning around the CTCF-binding sites, leading to an increase in nuclease accessibility around the CTCF-binding sites. **(C)** Association between SMARCA4/p68/p72 complex and CTCF. This complex is located on CTCF-binding sites around some TSS enriched with active histone marks. The p66/p72 complex is involved in promoting the assembly of transcription initiation complexes (TIC). The association between SMARCA4/p68/p72 suggests that this complex may be an important CTCF co-factor in chromatin architecture maintenance at some sites which is important for correct transcriptional output. **(D)** Association of architectural proteins with CRCs in *Drosophila*. At some insulator sites, as Fab-8, ISWI CRCs (which are directed to these sites by CP190) promote an open chromatin structure at dCTCF-binding sites for insulator function. ISWI depletion alters nucleosome phasing at these sites triggering a closed chromatin state, impairing dCTCF binding and insulator function.

Recent studies have also highlighted the importance of chromatin organization in the vicinity of the CTCF-binding sites. Specifically, TAD boundaries often contain several CTCF motifs, which in turn arrange the TAD boundary structure in a specific 3D nucleosome organization (Clarkson et al., 2019). SNF2H and SNF2L enzymes have an important role in regulating the nucleosomes at these regions. SNF2H depletion leads to loss of CTCF and there is an increment in nucleosome occupancy over the CTCF-binding sites (Figure 5A). CTCF recruits cohesin at most sites, therefore, depletion of SNF2H also leads to a reduction of cohesin at these sites (Wiechens et al., 2016).

These experiments suggested that SNF2H affects cohesin loading at a subset of CTCF sites by affecting nucleosome spacing. It was also established that SNF2L functions to maintain nucleosome organization of nucleosomes surrounding the CTCF sites, and it does so as part of the nucleosome remodeling factor (NURF) complex. Another interesting observation was that depletion of SNF2H affected the distance between the nucleosomes by causing an average increase of 25 bp. The opposite effect was observed for SNF2L depletion, in which case the distance between nucleosomes was reduced by 10 bp around transcription factor binding sites (Figure 5A) (Wiechens et al., 2016). Depletion of both enzymes also led to several changes in gene transcription. These effects, although seen at the nucleosome level, suggested that chromatin remodelers were also involved in higher order chromatin structure (see next section).

Another ATPase found to affect nucleosome positioning around the CTCF-binding site is SMARCA4 (SWI/SNF related, matrix associated, actin dependent regulator of chromatin, subfamily a, member 4, also known as Brahma related gene-1, BRG1). It is one of the ATPases of the SWI/SNF complex. SMARCA4 has been shown to regulate interchromosomal interactions between tissue-specific promoters during myogenesis (Harada et al., 2015) and binds to poised enhancers in embryonic stem cells (Rada-Iglesias et al., 2011).

Hi-C experiments following the perturbation of the level of SMARCA4 using shRNAs showed an increase in both intra- and interchromosomal associations in the subtelomeric regions, placing SMARCA4 as a regulator of higher order structures at these regions of the genome. Additionally, SMARCA4 perturbation led to changes in nucleosome positioning around the CTCF-binding sites, leading to an increase in nuclease accessibility around the CTCF-binding sites, thus affecting the TAD border strength, and allowing further intra-TADs interactions. These results placed SMARCA4 as a regulator of higher order chromatin structure (Figure 5B) (Barutcu et al., 2016).

Later, another group identified SMARCA4 as a partner of CTCF (Marino et al., 2019). SMARCA4 has many chromatin partners, among them are the p68/p72 RNA helicases, which also co-immunoprecipitate with CTCF. The complex with p68 (also called DEAD box RNA helicase p68, DDX5), p72, steroid receptor RNA activator, and MyoD are involved in promoting the assembly of a transcription initiation complex at the MyoD promoter. This complex was immunoprecipitated with CTCF and identified by mass spectrometry.

Given the association of SMARCA4 with p68, and the roles in the maintenance of a subset of TAD boundaries, the role of SMARCA4 at sites shared by DDX5 and CTCF was examined. These sites were shown to include a subset of genome wide CTCF sites located around the TSS and associated with marks of transcriptionally active chromatin (Figure 5C). The data suggested that SMARCA4 is an important CTCF co-factor for maintaining the correct transcriptional output and the correct chromatin architecture (Marino et al., 2019).

Biochemical studies have identified members of the cohesin complex that associate with CRC. Isolation of human ISWI (SNF2H)-containing CRC revealed that RAD21 interacts directly with this ATPase, as well as with members of the NuRD complex. Furthermore, they were found to bind together to specific Alu-rich regions in the genome and the absence of SNF2h impaired the binding of cohesion to these sites. As Alu sequences are rich in CpG dinucleotides, DNA methylation state was also found to modulate the association of cohesin to these sites (Hakimi et al., 2002). This study is of significance since it demonstrated that CRC was needed for the binding of cohesin to the chromatin.

In *Drosophila*, remodelers are also associated with architectural proteins. RNAi screening was used to identify regulators of the enhancer blocking activity of the Fab-8 insulator, whose activity depends on both CP190 and dCTCF. This screening led to the identification of approximately 80 genes. Among them, there were several ATPases, particularly ISWI and CAF-1 (chromatin assembly factor-1) members of the NURF and dREAM (dimerization partner, RB-like, E2F and multi-vulval class B complex) remodeling complexes, and other subunits of these complexes. It was established that lack of ISWI leads to a change in nucleosome phasing at dCTCF-binding site, making these sites less accessible to MNase digestion. Furthermore, it was demonstrated that CP190 directs the binding of these ATPases to specific insulator sites promoting an open chromatin structure (Figure 5D) (Bohla et al., 2014). These data provide further evidence that different chromatin complexes cooperate to maintain a correct chromatin structure at certain chromatin sites.

Several studies have identified the formation of loops between insulators and promoters (Erokhin et al., 2011). The formation of these loops promotes the binding of members of the basal transcriptional machinery, such as TFIID, by bringing together CRCs and histone modifying activities, such as the ones mentioned earlier in this review, and supports basal transcription of a number of genes. Interestingly, some insulators are found at the 3’ and 5’ UTRs of *Drosophila* genes, thereby promoting recycling of Pol II and control of gene transcription (Bushey et al., 2009; Nègre et al., 2010).

In *Drosophila*, as mentioned in the section “Introduction,” several architectural proteins interact with the components of CRCs, such as the DNA replication-related element binding factor (DREF). DREF is a transcription factor that binds to the DNA motif 5′-TATCGATA-3′ in the core promoter element (Matsukage et al., 2008). This motif is known as the DNA replication-related element (DRE). DREF physically interacts with the carboxy terminal of the ATPase, dXNP (an ortholog to the ATRX mammalian protein). This interaction negatively regulates the expression of genes, such as *pannier*, which are important for the correct development of the organism (Valadez-Graham et al., 2012). The role of DREF at the TAD boundaries is still unknown, however, it was shown that DREF can compete with BEAF-32 for its DNA recognition site (Hart et al., 1999). BEAF-32 has emerged as an important protein in the TAD boundaries and like the other architectural proteins, it would be interesting to identify their individual roles at these sites and their dependence on CRC such as dXNP (Ramírez et al., 2018).

### CRC in the Control of Compartments and TADs

Another subunit of the SWI/SNF complex shown to have a role in higher order chromatin structure is ARID1A. ARID1A is the largest subunit of this complex and belongs to the family of mammalian proteins known as “ARID,” because they were first identified to bind to AT-rich DNA elements. However, it is now accepted that not all the members of the family share this characteristic. All the members of this family are transcriptional regulators that are involved in many cellular processes such as cell differentiation, cell proliferation and development (Lin et al., 2014).

ARID1A plays an important role at the enhancer regions. ChIP-seq experiments using ovarian cancer cell-lines demonstrated that more than 80% of the peaks are localized at the enhancers and promoters, and that ARID1A co-localizes with a subunit of the condensin complex II called NCAPH2, which has recently been shown to associate with the shelterin protein, TRF1, and regulate telomeric stability (Wallace et al., 2019). ARID1A knockout affected the binding of NCAPH2 at H3K27Ac-marked enhancers genome-wide. The loss of binding occurred at almost 50% of the sites, but 12% of the sites showed an enrichment of CAPH2.

**FIGURE 6:**
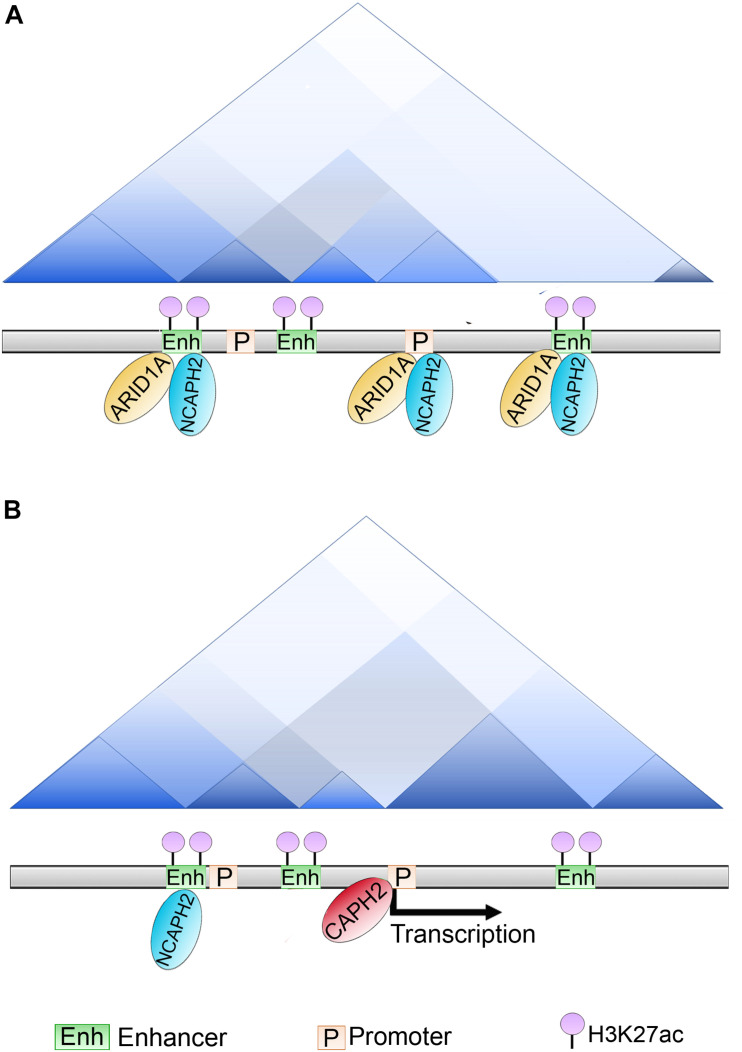
Role of ARID1A in compartments and TADs establishment. **(A)** In ovarian cancer cell lines ARID1A is present at enhancers and promoters co-localizing with the condensin subunit NCAPH2 (that recognizes and binds to H3K27ac marked enhancers). **(B)** Upon ARID1A depletion, NCAPH2 relocalizes to a subset of promoters. CAPH2 re-localization induces the gene expression of these promoter targets, whether this activity is responsible for B to A compartment switching is still unclear.

Some of them were at enhancers at which CAPH2 was relocalized and this relocalization induced gene expression of their promoter targets. Examination of the effect of ARID1A knockout on TAD formation showed that loss of ARID1A strengthened the TAD borders. This result indicates that ARID1A normally antagonizes the insulation of TADs. Additionally, when the delocalized higher order chromatin structure was analyzed at the level of compartments, there were 57 B-to-A switched compartments following ARID1A knockout (Figure 6). This result suggests that binding of ARID1A contributes to B compartment formation at these cells. Further, an overall decrease in interchromosomal interactions was observed suggesting that both NCAPH2 loss from ARID1A binding sites and *de novo* gain of binding sites contribute to changes in spatial chromosome partitioning following ARID1A inactivation (Wu et al., 2019).

Recently, another group demonstrated that clones of mouse ES cells with deletion of exon 6 of Smarca5/Snf2h (performed using CRISPR/Cas9) are still able to form ES colonies and show normal morphology. Two thousand differentially expressed genes were identified following Snf2h loss, which led to reduced proliferation and differentiation potential. Analysis of nucleosomal phasing using ATAC-seq (Active Transposase Accessibility Assay) and MNase-seq (Micrococcal Nuclease accessibility assay) revealed that the regulatory regions, but not TSS, were affected.

Importantly, nucleosome repeat length (which is the distance between the centers of neighboring nucleosomes and that allows one to determine the changes in the length of the linker DNA between nucleosomes) revealed an increment of 9 bp in the absence of Snf2h, which was specific to the depletion of this gene since SMARCA4 depletion had no effect on nucleosome repeat length (Beshnova et al., 2014). In addition, several transcription factors binding sites were analyzed and the CTCF DNA binding sites showed higher nucleosome occupation and DNA methylation following Snf2h depletion. Interestingly, when CTCF occupancy at this site was analyzed, CTCF levels were hardly affected. Nevertheless, Hi-C experiments showed that SNF2H depletion led to reduced insulation at the TAD boundaries. Furthermore, Hi-ChIP assays of Smc1, a component of the cohesin complex, demonstrated loss of Smc1 mediated loops (Barisic et al., 2019).

In summary, these results show that SNF2H impacts chromatin at the nucleosome level by changing nucleosome phasing, promotes changes in DNA methylation, and affects transcription factor binding. Additionally, this loss also affected the loop formation and TAD insulation. However, unlike in the case of ARID, mentioned above, it does not affect chromatin compartmentalization.

Other activities that are important are those of the topoisomerases. These enzymes are recruited by ATPases to these sites and contribute to the maintenance of the strength of the borders (Uusküla-Reimand et al., 2016). For instance, SMARCA4 ATPase activity is required for the recruitment of both Topoisomerase I and II (TOP I and TOP2A, respectively) to chromatin (Husain et al., 2016; Barutcu et al., 2017).

### CRC Association to Methyl CpG Binding Proteins

DNA methylation is recognized as a heritable epigenetic modification. Proteins from the methyl-binding domain (MBD) group, recognize these modifications and recruit several enzymatic activities to the specific DNA region, such as histone modifications and chromatin remodeling activities (Qian et al., 2015). All the proteins of this group have the conserved MBD domain. Some of them participate in transcriptional activation or repression by recruiting different enzymatic activities (Baubec et al., 2015). They are also involved in DNA repair, epigenetic maintenance coupled to DNA replication and histone deacetylation, and capable of promoting chromatin looping as explained below.

**FIGURE 7:**
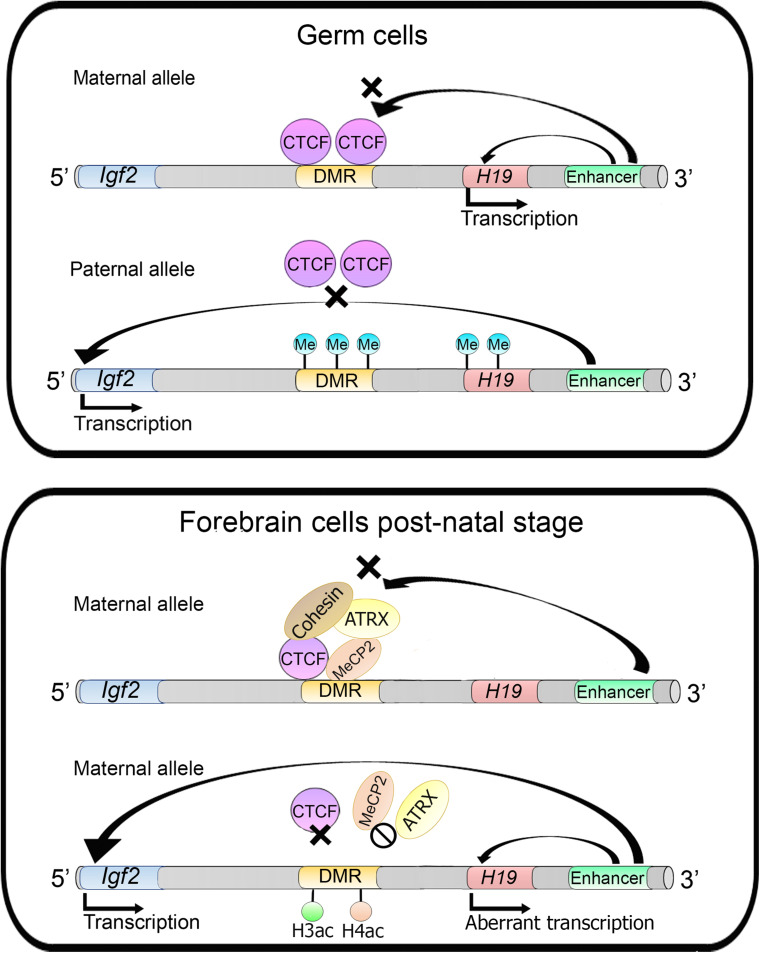
ATRX activity regulates imprinted genes. In germ cells into the *Igf2*/*H19* locus, *Igf2* gene is silenced through insulator establishment by CTCF and only the *H19* gene is expressed in maternal alleles. Whereas in the paternal allele the DMR is methylated preventing CTCF binding and impairing insulator activity allowing *Igf2* transcription. On the other hand, in forebrain cells of postnatal mice, in the maternal allele, the *H19* gene is silenced. MECP2 directs ATRX and cohesin recruitment to the DMR. MECP2 or ATRX depletion increases histones H3 and H4 acetylation which lead to changes in nucleosome occupancy and CTCF binding, causing an aberrant transcription of the *H19* gene.

In imprinted genes, only one parental allele is expressed whereas the other one is silenced. This silencing is controlled by a mechanism that includes an interplay between several proteins and DNA methylation. In the *Igf2/H19* imprinted genes, CTCF binds to the DMR (differentially methylated region) upstream of the *H19* gene on the maternal allele. Several enhancers are located downstream of the *H19* gene, while the *Igf2* gene is located further upstream of the DMR. CTCF binding at DMR acts as an insulator with enhancer blocking activity, preventing *Igf2* activation from the maternal downstream enhancers, which in turn, can only activate the *H19* gene. On the paternal allele, the DMR is methylated and CTCF does not bind to this region. Therefore, the enhancers can activate the *Igf2* gene (Figure 7). This mechanism seems to be different in germ cells and somatic cells (Lewis and Murrel, 2004).

In mouse forebrain cells MeCP2 directs ATRX (which is a member of SWI/SNF CRC) to the DMR that acts as an imprinting control region (ICR). MeCP2 binds specifically to the maternal allele along with cohesin and ATRX, and this complex favors CTCF occupancy at the DMR avoiding *Igf2* activation. MeCP2 or ATRX inactivation triggers the aberrant expression of the *H19* gene (at the post-natal stage) and an increase of histones H3 and H4 acetylation levels in the DMR. ATRX deficiency promotes a decrease of cohesin and CTCF occupancy at the DMR region, indicating that ATRX is necessary for cohesin and CTCF occupancy at these sites (Figure 7) (Kernohan et al., 2010). Moreover, it was later described that ATRX promotes long-range chromatin interactions between the DMR and different enhancers that direct *Igf2* expression. These chromatin loops are lost upon ATRX depletion and lead to an aberrant transcription of the *H19* gene (Kernohan et al., 2014).

Another example of the crosstalk between DNA methylation and chromatin remodelers in the maintenance of a chromatin loop was also studied in a model of low-grade astrocytoma in which the authors introduced a mutation in the isocitrate dehydrogenase enzyme in human neural stem cells (Modrek et al., 2017). Somatic mutations of this enzyme have been associated to this type of cancer and to changes in DNA methylation. Also, mutations in P53 and the chromatin remodeler ATRX have been identified in this type or tumors, damaging these three genes lead to a block in differentiation, abnormal DNA methylation at CpG regions which bear CTCF binding sites. The methylation at these sites impeded CTCF binding and, specifically at the SOX2 gene it affected the formation of a chromatin loop which is important for the transcriptional activation of this gene by enhancers which are positioned 700 kb away. All these mutations conform three “hits” necessary for tumor progression and invasiveness. The specific role of ATRX in the maintenance of this tumor phenotype is still not well understood, but it provides another evidence of this ATPase’s role in the maintenance of chromatin loops (Modrek et al., 2017).

## Discussion

The aim of this review was to present a global picture of the current research on CRCs and their role in maintaining higher order chromatin structure. The development of new techniques such as Hi-C and high throughput sequence have allowed the visualization of other levels of chromatin compaction in conjunction with mutations or lack of subunits of CRCs.

It is becoming clearer that CRCs’ subunits affect chromatin structures at different levels, whether it is affecting nucleosome phasing, impeding the union of transcriptional and architectural factors to DNA, modulating chromatin loops and some even modulating TAD insulation and higher order chromatin compartmentalization. Moreover, evolution has conserved many of these activities, even though loss of the orthologs display different phenotypes (such as the case of ISWI in *Drosophila* and mammals and the differences observed in nucleosome phasing), the global outcome, such as loss of architectural protein binding (for instance, CTCF) and insulation is the same. These data indicate that different species may use different strategies to achieve a correct control of chromatin organization.

CRC’s carry different enzymatic activities and although the current reviewed research has focused mainly on the ATPase activity, we can expect that other activities may also be involved in the control of higher order chromatin structures. Also, other physical properties which may also be promoted by CRCs such as liquid-liquid phase separation, will be worth studying. The relation of CRCs and higher order chromatin dynamics will shed light on different biological processes and enrich our understanding of these important complexes in development and disease.

## Author Contributions

Both authors contributed to the article and approved the submitted version.

## Conflict of Interest

The authors declare that the research was conducted in the absence of any commercial or financial relationships that could be construed as a potential conflict of interest.
